# Primary Leiomyosarcoma of the Pancreas—a Case Report and a Comprehensive Review

**DOI:** 10.1007/s12029-016-9872-y

**Published:** 2016-09-09

**Authors:** Jon Arne Søreide, Erling Sandøy Undersrud, Mohammed S. S. Al-Saiddi, Tore Tholfsen, Kjetil Søreide

**Affiliations:** 1 Department of Gastrointestinal Surgery, Stavanger University Hospital, POB 8100, N-4068 Stavanger, Norway; 2 Department of Clinical Medicine, University of Bergen, Bergen, Norway; 3 Department of Pathology, Stavanger University Hospital, Stavanger, Norway; 4 Department of Radiology, Stavanger University Hospital, Stavanger, Norway

**Keywords:** Pancreas, Leiomyosarcoma, Mesenchymal tumor, Review

## Abstract

**Purpose:**

Primary mesenchymal tumors of the pancreas are rare, with leiomyosarcomas the most encountered entities among the pancreatic sarcomas. With few exceptions, single case reports published over the last six decades constitute the entire scientific literature on this topic. Thus, evidence regarding clinical decision-making is scant.

**Methods:**

Based on a case report and an extensive literature search in PubMed, we discuss the clinical aspects and current management of this rare malignancy.

**Results:**

We identified only two papers with more than a single case presentation; these institutional patient series were limited to five and nine patients. Additionally, a few papers sought to summarize the individual case reports published in the English and/or Chinese language. The clinical presentation is rather non-specific. Moreover, modern imaging modalities are insufficiently accurate to diagnose leiomyosarcoma of the pancreas. Treatment goals include a complete resection with free margins. Proper morphologic examination using immunohistochemistry and the application of a grading system are clinically important for prognostication. The efficacy of adjuvant treatments has not been established.

**Conclusion:**

Primary pancreatic leiomyosarcoma is extremely rare, and the scientific literature is primarily based on single case reports. Conclusions on management and prognosis should be drawn with caution. A multidisciplinary team consultation is warranted to discuss a thorough individual treatment plan based on the available scientific literature, despite its low evidence level.

## Introduction

Pancreatic adenocarcinoma is a common and increasingly prevalent malignancy of the pancreas worldwide [1–3], representing more than 90–95 % of all pancreatic malignant tumors. In contrast, primary mesenchymal tumors of the pancreas are rare [4–7] with a reported 0.1 % incidence of pancreatic sarcoma diagnosed after autopsy involving 5057 cases of malignant pancreas tumors [8]. Most pancreatic mesenchymal tumors are gastrointestinal stromal tumors (GIST) or neurogenic tumors [4, 6]. Among the pancreatic sarcomas, leiomyosarcoma is the most encountered entity [9]. Only 69 cases were reported in scientific English (*n* = 49) and Chinese (*n* = 20) medical journals [10] from 1951, when Ross [11] reported the first case of a primary pancreatic leiomyosarcoma (PLMS), to 2013. Thus, the clinical presentation and diagnostic characteristics of this rare lesion may be difficult to anticipate.

In this study, we present a case report and aim to discuss the clinical aspects and current management of this rare malignant pancreatic tumor based on a comprehensive review of the available literature.

## Case Report

**Fig. 1:**
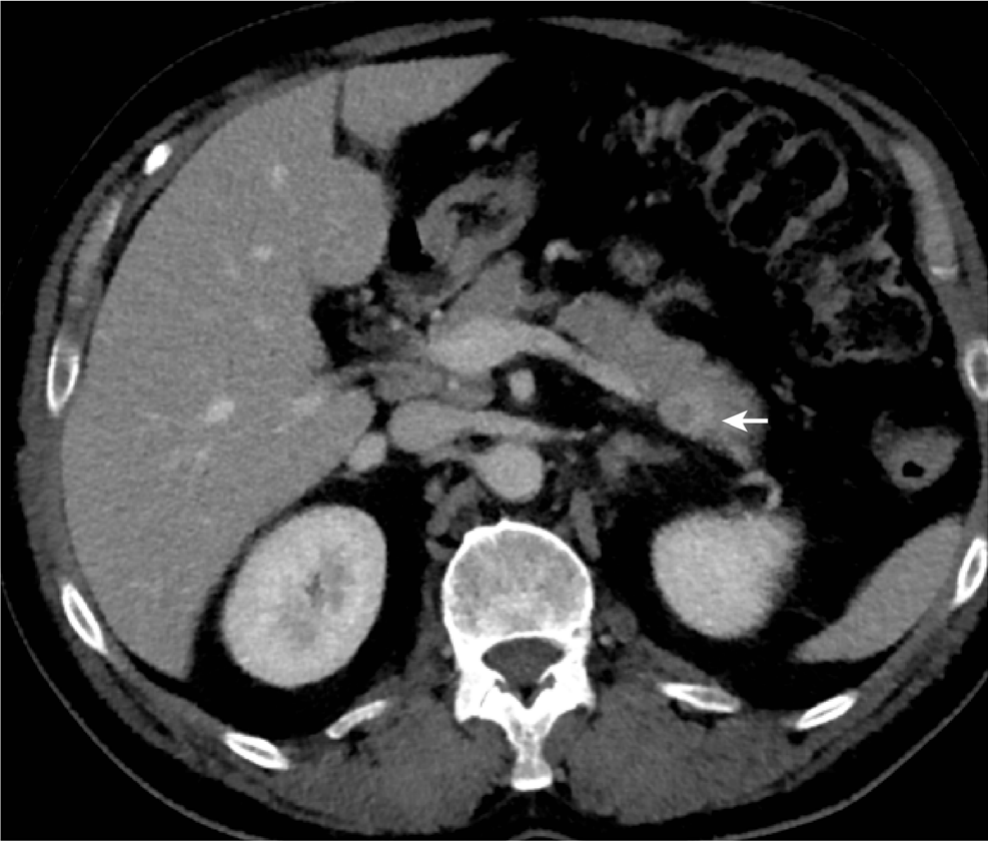
Abdominal computed tomography (*CT*) shows a 15 × 12-mm tumor (*white arrow*), which is heterogeneously enhanced by contrast media

**Fig. 2:**
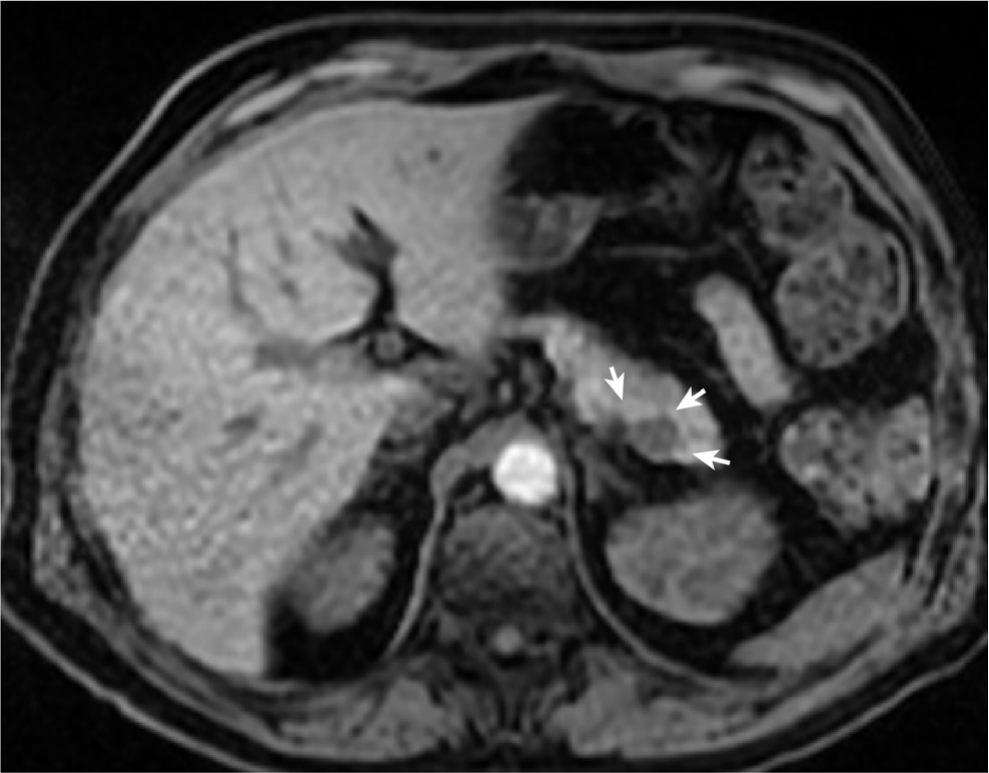
Magnetic resonance imaging (T1 sequence) shows the same lesion identified by CT as a 17 × 14-mm heterogeneously enhanced lesion (*white arrows*) of the pancreatic tail

A 55-year-old male with a previous history of hepatitis C and a history of two cardiac infarctions was seen by his general practitioner in evaluation of epigastric symptoms. A general work-up that included a negative upper endoscopy was performed. An abdominal computed tomography (CT) performed outside the hospital revealed a lesion within the tail of the pancreas **(**Fig. 1 **)**, which was also visualized on an additional magnetic resonance tomography imaging (MRI) exam (Fig. 2). Because the imaging findings suggested a heterogeneously enhanced lesion, a neuroendocrine tumor was a differential diagnostic consideration.

Based on the imaging findings, the patient was referred to our department. The general laboratory biochemistry analysis was within normal limits. Except for a slightly increased serum chromogranin A (CgA) of 6.7 nmol/l (normal, <3.5 nmol/l), other tumor markers were normal including carcinoembryonic antigen (CEA) (2 μg/l; normal, <5 μg/l) and carbohydrate antigen 19–9 (CA 19–9) (5 kU/l; normal, <60 kU/l).

**Fig. 3:**
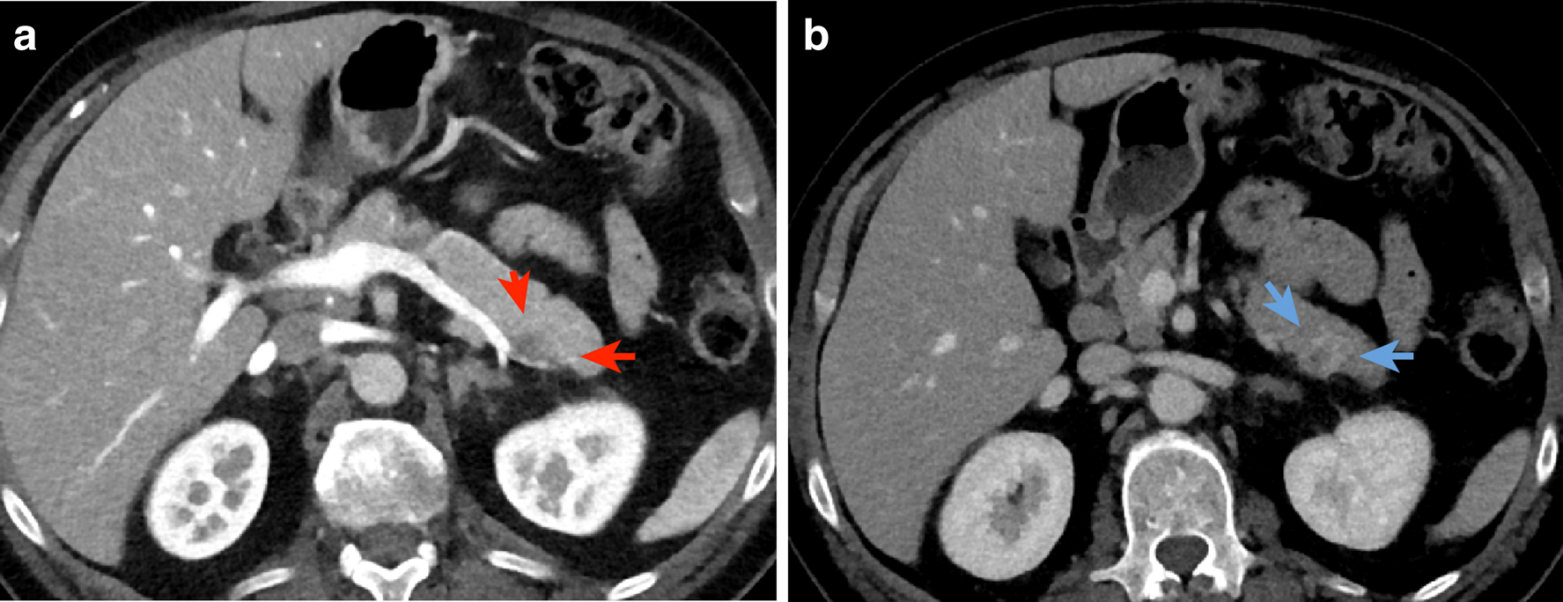
**a** CT of the pancreas with intravenous contrast in the arterial phase (*red arrows*) shows a 17 × 15-mm tumor with heterogeneous contrast enhancement. **b** CT of the pancreas in the venous phase shows the tumor (*blue arrows*) in the pancreatic tail

Three-phase pancreas CT protocol imaging was performed, showing a 17 × 15-mm solid lesion that was slightly abutting against the splenic vein in the pancreatic tail **(**Fig. 3 **).** Clinical symptoms or signs of metastatic disease or a functional pancreatic neuroendocrine tumor were absent, as were any imaging findings of metastatic disease.

After a multidisciplinary team consultation (i.e., surgeon, oncologist, radiologist), primary surgical treatment of the suspected malignant tumor was recommended. With informed consent from the patient, an uneventful open distal pancreas resection with splenectomy was performed. The patient developed postoperative pneumonia, and eventually a wound rupture occurred that prompted re-operation on the ninth postoperative day (POD). He was discharged home on the 13th POD after the pancreas resection in good condition, and his subsequent recovery was uneventful.

Macroscopically, a 17-mm homogenous tumor with a solid, slightly whorled cut surface was identified in the distal pancreas in close proximity to and probably originating from a venous blood vessel. On the dorsal pancreatic surface of the specimen, a small inked focus was seen on a part of the tumor margin. While this tissue area was somewhat traumatized, which would likely blur the inking, the other assessable surgical margins were tumor free, suggesting a radical resection (R0).

**Fig. 4:**
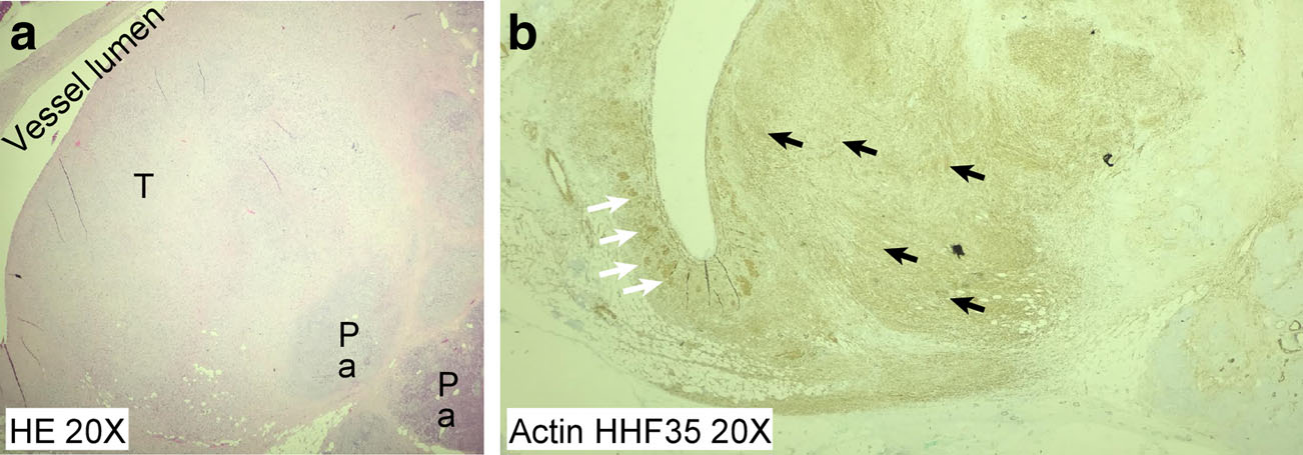
**a** HE ×20 magnification. Lumen of the intrapancreatic venous vessel with the tumor (T) infiltrating the pancreatic tissue (Pa). **b** Positive staining with actin HHF35. *White arrows* indicate smooth muscle in the vessel wall. *Black arrows* indicate the spindle cell proliferation as positive for muscle-specific actin, which is consistent with a leiomyosarcoma

**Fig. 5:**
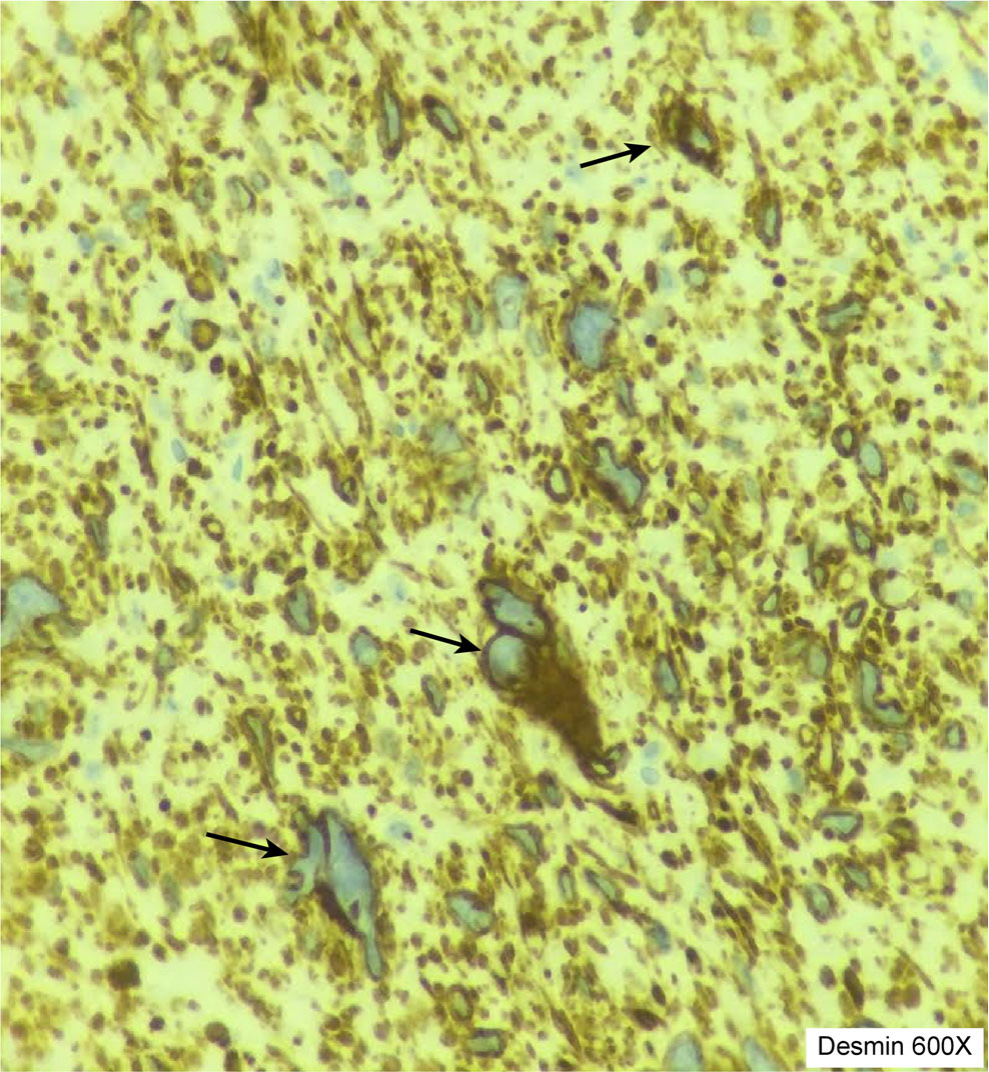
Discernibly atypical tumor cells (*white arrows*) with positive staining for desmin, a relatively specific marker for muscle differentiation (magnification ×600)

**Fig. 6:**
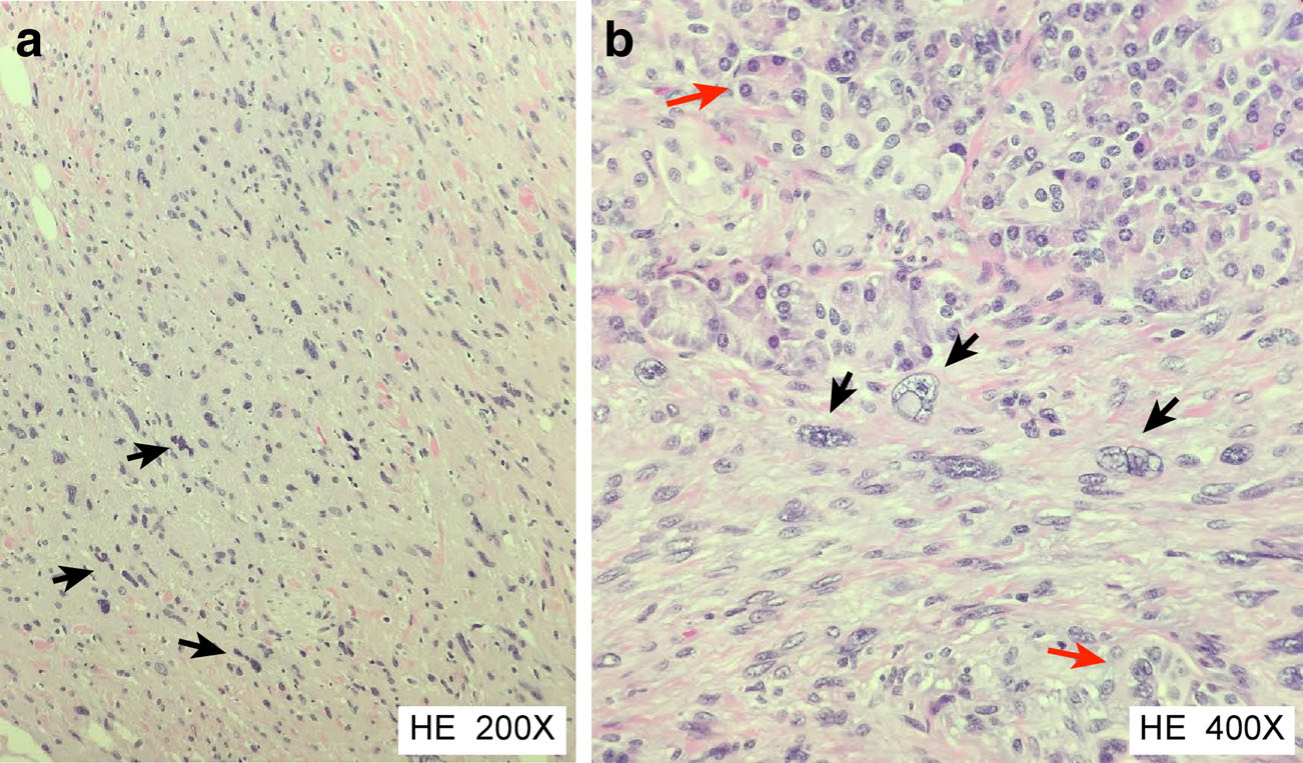
**a** Discernibly atypical sarcoma cells (*black arrows*), some of which are pleomorphic and show abnormal nuclei, infiltrating the pancreatic soft tissue (magnification ×200). **b** Normal acinar pancreatic parenchyma (*red arrows*) with atypical sarcoma cells infiltrating the pancreatic tissue (*black arrows*) (magnification ×400)

Microscopy confirmed the close relationship of the tumor to a venous blood vessel (Fig. 4) and revealed an infiltrating tumor with spindle cell morphology emanating from the vessel wall. Cellular atypia was evident, and mitoses were easily identified (Fig. 5). Atypical mitoses were also present (Fig. 6). Immunohistochemical staining was positive for actin 1A4 (smooth muscle actin), muscle-specific actin (actin HHF35) (Fig. 4b), and desmin (Fig. 5), supporting smooth muscle differentiation. No staining for CD117 and CD34 or neuroendocrine markers (synaptophysin and chromogranin A) was evident. The proliferation marker Ki-67 was positive in approximately 50 % of the tumor cells in “hot spots” (i.e., the strongest positive area of the tumor).

A routine section taken from the spleen was negative for tumor markers. A total of five benign lymph nodes were harvested.

The tumor morphology was consistent with a leiomyosarcoma of the pancreas originating from an intrapancreatic venous blood vessel [12]. According to the French grading system [13, 14], which is a three-grade system mainly based on histologic type and subtype, tumor necrosis, and mitotic activity, the tumor was grade II (Table 1).

**Table 1:** Criteria definitions and grading system for soft-tissue sarcomas according to “the French system” (Fédération Nationale des Centers de Lutte de Cancer, FNCLCC) (after Trojani et al. [14])

Criterion	Score	Definition
Tumor differentiation	1	Well-differentiated tumors
	2	Sarcomas with specific histologic typing
	3	Embryonal, undifferentiated, or sarcomas of uncertain tumor type
Mitosis count (counted at ×400 magnification in ten consecutive fields)	1	0 to 9 mitoses per 10 HPF^a^
	2	10 to 19 mitoses per 10 HPF
	3	More than 20 mitoses per 10 HPF
Tumor necrosis	0	No necrosis on any examined slides
	1	*<*50 % tumor necrosis
	2	≥50 % tumor necrosis
Histologic grade		
Grade I	Total score 2–3	
Grade II	Total score 4–5	
Grade III	Total score 6–8	

^a^A high-power field (HPF) is equal to 0.1734 mm^2^

## Literature Search

A literature search in PubMed was conducted using terms including “pancreas,” “pancreatic,” “sarcoma,” “leiomyosarcoma,” “angioleiomyosarcoma,” and “mesenchymal.”

## Results

We were unable to identify any scientific publications on this topic since 2015, when Milanetto et al. reported another single case and summarized 44 previous papers published between 1951 [11] and 2014 [6] in the English literature, including only 2 reports comprising more than a single case (i.e., 5 [8] and 9 patients [9]). Except these few reports [8, 9], the literature published over the last six decades on this topic comprises single case reports only.

### Brief Summary of the Literature

Baylor and Berg [8] summarized their experience of 5 cases identified at autopsy among 5000 deceased patients with presumptive pancreatic cancer, whereas Zhang et al. [9] reported on an institutional series of 9 patients diagnosed at the Mayo Clinic, MN, USA from 1994 to 2006. A review of 35 cases of pancreatic leiomyosarcoma in the English scientific literature was reported by Aihara et al. in 2002 [15]. A decade later (in 2013), a systematic review of 69 case reports published in Chinese and English journals summarized pertinent information and described the clinical characteristics and the prognosis of this rare entity [10]. This report included a search for case reports via the China Knowledge Resource Integrated Database. Furthermore, in 2015, Milanetto et al. [16] summarized the clinical features of 45 cases reported in the English literature up to that date, which included their own case.

In summary, based on the available literature on this topic [8–10, 15, 16], the distribution between the sexes is equal, and the median age is approximately 55 years (almost two decades younger than for pancreatic ductal adenocarcinoma), ranging from 15 to 85 years. Clinical symptoms were reported in 90 % of cases, with the presence of an abdominal mass (50 %), abdominal pain (43 %), and weight loss (33 %), the most common clinical symptoms encountered in these patients [10]. Jaundice, anemia, gastrointestinal bleeding, and vomiting were also reported in a few patients. Whereas seven of nine tumors were located in the head of the pancreas in the Mayo Clinic series [9], no differences regarding the tumor location, i.e., in the head or the body-tail of the pancreas, were determined from the available literature [10]. At diagnosis, a median tumor size of 10 cm (range, 1–30 cm) was reported.

Based on the available information on gross morphology, 53 % of the tumors were solid, 16 % were cystic, and 31 % had a mixed pattern [10].

Zhang et al. reported that four of nine patients had liver metastases at the time of diagnosis [9]. Based on a review of 68 patients with reported metastatic status [10], distant metastasis was diagnosed in 25 % of the patients and 19 % had local invasion into adjacent vessels/organs. In contrast, lymph node metastasis was confirmed in only one patient (1.5 %) [10].

Information on surgical treatment was available for 62 patients: 65 % underwent radical surgery, whereas the remaining patients had a palliative procedure or a biopsy only. Long-term follow up data were available for survival analysis in 49 (71 %) of the 69 cases [10]. The overall 1-, 3-, 5-, and 10-year survival rates were 66.6, 51.2, 43.9, and 29.3 %, respectively [10]. Multivariate analysis showed that a non-radical resection was a significant adverse prognostic factor and that adjacent organ/vessel invasion might also be a detrimental factor for long-term survival [10]. In the Mayo Clinic series [9], a median survival of 13 (range, 5–98) months was achieved in the nine patients, of whom four were surgically treated with a pancreatoduodenectomy, three had a palliative procedure, and two underwent biopsy only but endured a detrimental outcome when a resection could not be employed.

Surgical resection with free margins was suggested as the only possible curative treatment by the authors, and a role for adjuvant therapies has remained undetermined [9, 10, 17].

## Discussion

Solid pancreatic tumors require a standard work-up including proper imaging [18, 19]. Initial suspicions favor ductal adenocarcinoma or less frequently, pancreatic neuroendocrine tumor [19–22]. In patients with a history of a malignancy, metastases to the pancreas from another primary site should also be considered [19, 23–25].

Pancreatic origin was likely in the present case, with a small tumor clearly confined to the pancreatic gland [26]. Moreover, metastatic leiomyosarcoma isolated to the pancreas from a distant primary tumor site is extremely rare [27].

The rare entity of primary pancreatic leiomyosarcoma may be diagnosed exclusively based on histopathologic morphology [28]. The term *leiomyosarcoma* includes a spectrum of diseases ranging from low-grade cutaneous lesions with a relatively indolent behavior to aggressive deep lesions of the abdomen or extremities with significant metastatic potential. Distinguishing a leiomyosarcoma from the most commonly encountered mesenchymal gastrointestinal tumor (i.e., the gastrointestinal stromal tumor, GIST [29], characterized by the presence of activating mutations in *KIT* or *PDGFRA*, and expression of CD 117 and/or CD34) is highly important. Characteristically, a leiomyosarcoma shows positive staining for smooth muscle actin and desmin, as also demonstrated in the present case, and may be properly diagnosed when relevant immunohistochemistry (IHC) is employed. The advent of molecular pathology and expanded immunohistochemical staining options have conferred greater accuracy to this diagnosis [28, 30]. Histologic grading [14] is generally an important prognostic factor and also an indicator of metastatic risk in adult soft tissue sarcomas [13]. The French [14] and the National Cancer Institute [31, 32] grading systems are the most commonly used, both of which are three-grade systems. Grading should be included routinely in the pathologic report [13].

Thus, while the rarity of pancreatic mesenchymal tumors is evident, misclassification between subclasses of other rather uncommon mesenchymal tumors may partly explain the extreme scarcity of primary pancreatic leiomyosarcomas reported in the literature. Moreover, the exact topographic localization of the reported leiomyosarcomas has not always been provided, which may also obscure the exact organ distribution [33].

As observed in the present case, the clinical signs and symptoms in this group of patients are non-specific [10]. In the absence of overt clinical manifestations, small pancreatic tumors are typically identified incidentally. Arriving at an accurate diagnosis based on imaging alone may be challenging [18, 19, 34]. This dilemma particularly obtains for the rare pancreatic tumors (i.e., solid pseudopapillary tumor (SPT), pancreatic lymphoma, pancreatoblastoma, and metastasis to the pancreas). Alternatively, Srivastava et al. [35], based on the CT findings of four patients, proposed that pancreatic leiomyosarcoma should be entertained when all the findings of large size at presentation, increased vascular enhancement, and the absence of biliary dilatation are present. A similar conclusion regarding a large heterogeneous mass containing necrotic and calcified areas has been reported by other investigators [36, 37]. As described by Machado et al. [37], an ^18^F-fluorodeoxygliose positron emission tomography (FDG-PET) scan revealed an area of increased tumor metabolic activity and a central area of low metabolic activity, which supported suspicions of a malignant mesenchymal tumor. Others have reported on contrast enhancements in both the arterial and venous phases [38]. According to Paciorek and Ross [39], tumor localization using MRI was best achieved through the unenhanced T1- and T2-weighted images. Consistent with the observed MRI characteristics of leiomyosarcomas occurring in other organs, most pancreatic lesions were isointense with skeletal muscle on T1-weighted images and hyperintense on T2-weighted images. Furthermore, gadolinium enhancement was usually heterogeneous. Pancreatic leiomyosarcomas had characteristics similar to typical pancreatic adenocarcinomas; however, diffusion-weighted imaging techniques contributed only marginally to the diagnosis, detection, and characterization of a focal pancreatic lesion. This study concluded that differentiation from the far more commonly occurring adenocarcinoma was not possible [39]. Conversely, as observed in pancreatic metastases from primary leiomyosarcomas located elsewhere, CT imaging displays a hypovascular pattern in the arterial phase, with homogeneous enhancement in the venous phase in half of the patients [40]; thus, multidetector CT angiography is regarded as a highly accurate technique for characterizing pancreatic metastases [41, 42].

While the tool box for pancreas imaging is continuously expanding and improving [18], still a number of common and uncommon pitfalls can be encountered [43]. By the clinical introduction of high-resolution endoscopic ultrasound (EUS), a new imaging approach became available. In addition to describing various patterns of the pancreatic gland, pathology related to its ductal structures or vessels, and any diagnosed lesions could be reported by this novel approach. Moreover, EUS enables a more imaging-directed biopsy of lesions, and also a novel path to procure core tissue for molecular analysis [44]. Thus, its use has gained broad acceptance in recent years, and has been part of the diagnostic and staging tools, both for solid and cystic primary pancreas tumors [45–47], but also for suspected metastasis to the gland [23]. Of note, EUS is operator-dependent, and specialized training in both endoscopy and ultrasound is required. High-quality EUS was not available at our institution at the time of diagnostic work-up for our patient. If available at that time, EUS with imaging-directed biopsy could have provided information of clinical relevance. In this particular case, with a rather small resectable pancreatic tumor, most likely the surgical treatment and timing, and the procedure employed would have been the same.

In retrospect, our interpretation of the peripheral contrast enhancement of the tumor together with a slightly elevated CgA as suggestive of a neuroendocrine tumor was incorrect. However, a neuroendocrine tumor (NET) or a neuroendocrine carcinoma (NEC) may be more common in the pancreas than a primary leiomyosarcoma, and a clear distinction between these entities may prove difficult without well-defined characteristics identified for each entity.

Surgery with free tumor margins is standard treatment for localized sarcomas [18]. Due to the tumor size encountered at the time of diagnosis, this procedure may include a major radical or perhaps a multivisceral resection in many patients. However, when the pancreatic tumor is small, as in the present case, limiting the extent of surgery to achieve free margins appears appropriate [15]. Indications for adjuvant treatments with radiation and/or chemotherapy in the setting of abdominal sarcomas are not well described, and no consensus exists regarding the clinical effects of these modalities. Currently, doxorubicin-based chemotherapy is suggested as first-line treatment in adults with leiomyosarcoma not amenable to curative-intent surgery [48], whereas 6 cycles of doxycycline and ifosfamide have been reported as an option in the adjuvant setting [49]. Moreover, gemcitabine-based chemotherapy has been evaluated, though inconclusively [50].

Recently, more attention has been directed toward the underlying biology of individual sarcoma subtypes. Additionally, greater specificity has been applied to the selection of chemotherapeutic agents based on their activity against the individual histological subtypes [51]. Despite these advances, the management of sarcomas, particularly concerning rare subtypes, remains a major challenge. With the paucity of available clinically effective treatments for patients with advanced or metastatic leiomyosarcoma, the recently entertained novel therapeutic targets may auger hope for the development of alternative therapeutic strategies [52].

The extremely rare occurrence of primary pancreatic leiomyosarcoma and a scientific literature primarily based on single cases present challenges in establishing any firm conclusions on management and prognosis. In patients with a pancreatic tumor, proper imaging should be employed to arrive at the most likely pre-operative diagnosis and to stage the disease properly. Moreover, radical surgery with free margins should be the aim when possible, and the proper use of relevant IHC should enable an accurate diagnosis. Whether molecular parameters may contribute to the tumor grading and prognostication remains undetermined [13]. In cases of primary pancreatic leiomyosarcoma, multidisciplinary team consultation is warranted, although the evidence for any decision-making rests on a rather sparse literature.
